# Editorial: Protecting the acutely injured lung: Physiologic, mechanical, inflammatory, and translational perspectives

**DOI:** 10.3389/fphys.2022.1009294

**Published:** 2022-09-06

**Authors:** Gary Nieman, Maurizio Cereda, Luigi Camporota, Nader M. Habashi

**Affiliations:** ^1^ Department of Suregy, Upstate Medical University, Syracuse, NY, United States; ^2^ Department of Anesthesia, Critical Care and Pain Medicine, Harvard Medical School, Boston, MA, United States; ^3^ Department of Adult Critical Care, Guy’s and St Thomas’ NHS Foundation Trust, Health Centre for Human and Applied Physiological Sciences, London, United Kingdom; ^4^ 1R Adams Cowley Shock Trauma Center, University of Maryland School of Medicine, Baltimore, MD, United States

**Keywords:** Intensive Care, Critical Care, ARDS, MODS, ECMO, treatments

## Introduction

**FIGURE 1:**
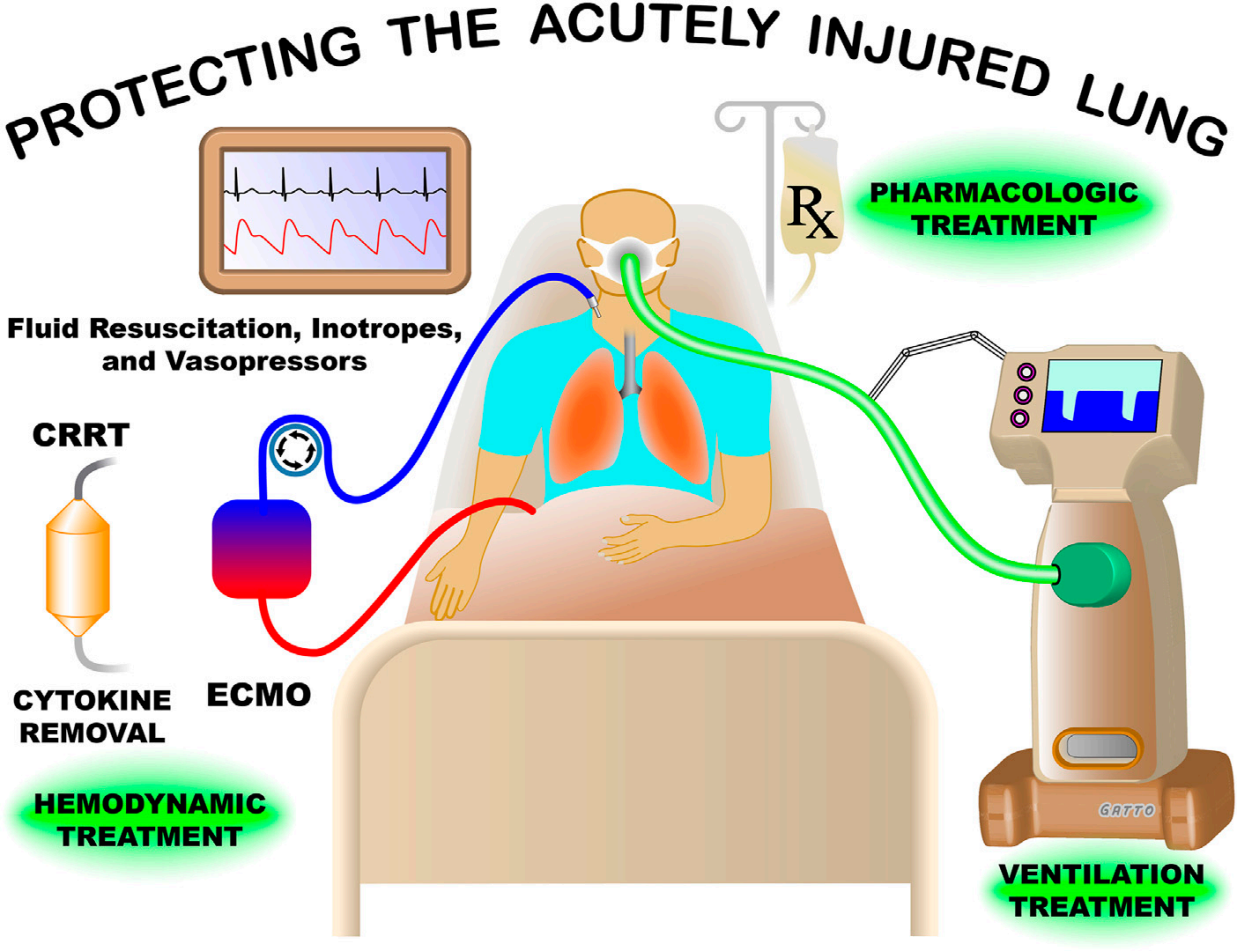
Treatment of the patient with acute lung injury (ALI) in the modern intensive care unit is extremely complex. Mechanical ventilation must be adjusted to maintain life supporting oxygenation and ventilation without causing an unintended ventilator induced lung injury (VILI). The impact of ventilation pressures must also be weight against the possible negative effect on hemodynamics including arterial blood pressure and cardiac output, which are supported with fluid resuscitation and vasoactive agents/inotropes. Currently, no pharmacologic treatments are available for the ALI patient, but development of drugs and gene therapy designed to reduce systemic inflammation, preserve air-blood barrier integrity, and remove edema from alveoli continues. Continuous renal replacement therapy (CRRT) can be used for acute kidney failure and can be combined with novel strategies to remove inflammatory cytokines and other mediator from the blood. Great advancements have been made in treating the ALI patients but much more work needs to be done, with the hope of significant breakthroughs in the near future.

Our Research Topic broadly reviewed multiple facets of treating the critically ill patient with acute lung injury (ALI) (Figure 1). There were nine papers discussing various aspects of protecting the acutely injured lung from ventilator induced lung injury (VILI), three papers covered inflammatory characteristics, three treatment strategy papers including veno-venous extracorporeal membrane oxygenation (vv- ECMO), gene therapy, and the physiology associated with successful and unsuccessful clinical trials on patients with ALI. Lastly, there was one paper reviewing lung fluid balance in the normal and acutely injured lung.

## Protective mechanical ventilation

Acute respiratory distress syndrome (ARDS) was first identified in 1967 but remains a significant medical problem today (Ashbaugh, Bigelow et al., 1967). Fifty-five years later, the only treatments for ARDS are supportive, mainly in the form of mechanical ventilation (MV) (Matthay, Zemans et al., 2019). Unfortunately, inappropriately set MV can inadvertently cause VILI, significantly increasing ARDS-related mortality (Del Sorbo, Goligher et al., 2017) and the quest for a ventilator mode and method to minimize VILI is still being intensely studied. (Pelosi, Ball et al., 2021).

Rola and Daxon conducted a case series on COVID-19 induced ARDS (CARDS) patients. They showed that the time controlled adaptive ventilation (TCAV™) method to set and adjust the airway pressure release ventilation (APRV) mode was highly effective at stabilizing and then gradually reopening the collapsed and edema filled lungs of CARDS patients. The TCAV™ method [developed by Nader M. Habashi](Habashi 2005) is both personalized and adaptive and is the most studied method to set and adjust APRV (Nieman et al.). TCAV uses inspiratory and expiratory time to rapidly stabilize and then gradually open collapsed tissue over hours or days (Kollisch-Singule et al.). They showed the efficacy of this *Stabilize Lung Approach (SLA)* approach in CARDS patients using chest X-rays, ventilator parameters, and arterial blood gases (ABGs). They also discussed physiologic mechanisms for TCAV efficacy, fine points of TCAV settings, and complexities of training an entire intensive care unit (ICU) team on TCAV™ use. Lastly, they discussed the problem of the “apparent cure” when TCAV™ has a patient’s lung fully recruited with near normal ABGs and respiratory system compliance (C_RS_) such that they no longer meet the Berlin defined ARDS. If these patients are converted back to conventional mechanical ventilation (CMV) the lungs may rapidly re-collapse. (Nieman, Andrews et al., 2018). They presented a case in which a CARDS patient with a TCAV™-induced open lung (“apparent cure”) was converted back to CMV and the lungs quickly derecruited.

APRV is controversial due to many publications discussing the harmful components of the mode without scientific support. Andrews et al. published an extensive review of 10 APRV Myths and Misconceptions, which are debunked using significant supporting scientific literature. Some of the discredited myths discussed include APRV can cause barotrauma, generates high tidal volumes, and creates unsafe auto-PEEP. The paper closes with a discussion on misconceptions dealing with APRV clinical trials. By exposing the truth concerning the physiologic impact of APRV, clinicians may better understand the mode leading to more effective use and improved patient care.

Suarez-Sipmann et al. used expired CO_2_ kinetics to personalize lung protective ventilation for ARDS patients. Volumetric capnography (Vcap) that represents the volume of expired CO_2_ in one signal breath can inform clinicians about pulmonary perfusion, end-expiratory lung volume, dead space and ventilation inhomogeneities. Recent work has shown that Vcap can possibly be used to continuously measure end-expiratory lung volume (EELV), lung strain, and effective pulmonary blood flow. The ability to measure all of the above at the bedside would be an incredible tool in the clinicians’ treatment options. Using these parameters, the clinician can modify the ventilator mode and the method necessary to maximize EELV and minimize lung strain that is individualized to each patient. With future development Vcap might be able to optimize lung protective ventilation strategies and reduce VILI-related mortality.

Most protective ventilation research efforts use CMV modes adjusting tidal volume (V_T_) and positive end expiratory pressure (PEEP) with and without recruitment maneuvers (RM), in an attempt to reduce VILI. Ismaiel et al. used CMV in a rat ARDS model to differentiate the pathophysiologic impact of mechanical and inflammatory injury as VILI mechanisms. They showed that low tidal volume (LV_T_) strategy reduces VILI by limiting mechanical damage, while hypercapnia limits pro-inflammatory and biochemical mechanisms of injury. They close by suggesting that LV_T_ combined with hypercapnia may work synergistically to reduce VILI.

A review of protective MV for the pediatric ARDS (PARDS) patient by Kollisch-Singule et al. discussed both traditional and novel modes of MV including APRV, high frequency oscillatory ventilation (HFOV), high frequency percussive ventilation (HFPV), and high frequency jet ventilation (HFJV). Unfortunately, results of this review show no consistent outcomes among modes, nor does it identify an optimal ventilator mode or method for the PARDS patient. They concluded that only high quality randomized controlled trials (RCTs) would be able identify if an optimal ventilation strategy does exist for the PARDS patient but caution that these trials are very difficult to conduct.

An extensive review of the HFOV mode was conducted by Miller et al. The review covers HFOV theory of operation, mechanics, and characteristics of all ventilators that can deliver the mode. Evidence of HFOV efficacy from bench models, animal studies and in both adult and pediatric patients are reviewed in detail. Although the physiologic rationale for HFOV is sound with many positive animal studies, the mode has lost popularity with both adult or pediatric patients following the failed 2013 OSCAR and OSCILLATE clinical HFOV RCTs (Ferguson, Cook et al., 2013; Young, Lamb et al., 2013). Possible reasons for the poor outcomes are discussed. There is continued study using HFOV in pediatric patients but the current RCTs are low quality. Since the mode is difficult to use it was suggested that staff education and competency are critical in future study.

In a crossover clinical trial in 20 patients with mild to moderate ARDS, Ball et al. studied the impact of multiple levels of variable pressure support ventilation (vPSV) on short term lung function. It has been shown that vPSV, compared with PSV, can improve oxygenation and patient-ventilator synchrony, but these studies were conducted using only one variability level at a fixed pressure support. In this study the addition of multiple levels of variability did not improve oxygenation and high variability levels increased patient-ventilator synchrony.

Accurate measurements of lung compliance (C_L_) and driving pressure (ΔP) are critical when managing ventilator settings on patients with ARDS, which are often measured clinically using plateau pressure (Pplat). Although this ‘stop flow’ condition is valuable and universally accepted, it may underestimate the maximum stress that occurs in lung tissue under dynamic conditions. Tawfik et al. compared the static measurement of C_L_ and ΔP with another static method and two dynamic compliance measurement methods. The other static method to calculated C_L_ and ΔP the pressure at zero flow and the two dynamic methods used the inspiratory slope during inflation with a constant flow and the expiratory time constant method. They found that the static measurement using Pplat may underestimate the maximum pressure exposed to lung tissue during dynamic inflation. Whereas the static method using zero flow and the dynamic method using inspiratory slope gave a truer estimate of maximum pressure exposed to alveoli. This pilot study suggests further studies are necessary to identify the optimal method to measure C_L_ and ΔP necessary to improve patient outcomes.

Mechanical ventilation for patients with ALI can lead to ventilator induced diaphragmatic dysfunction (VIDD) (Vassilakopoulos and Petrof 2004). Currently, ultrasound and invasive phrenic nerve stimulation are the only bedside tools available to monitor diaphragm function and VIDD. Spadaro et al. measured fast and slow isoform of troponin I (fsTnL and ssTnl, respectively), which are specific markers of skeletal muscle damage. The goal was to identify the trend of skeletal troponin during weaning and compare it with fsTnL and ssTnl levels with diaphragmatic ultrasound in healthy volunteers. They found that fsTnL and ssTnl have specific and different trends during weaning with the fsTnL decreasing during the early phase of weaning, while high initial values of ssTnl were correlated with a larger diaphragmatic displacement over time. More work is necessary to identify if these markers can be used to identify diaphragm function in ALI patients.

## Inflammation and biotrauma

There are three major mechanisms of VILI: Atelectrauma (alveolar recruitment/derecruitment–R/D), volutrauma (alveolar overdistension), and biotrauma (excessive release of inflammatory mediators caused by atelectrauma and volutrauma). Dysregulation of the renin-angiotensin system (RAS) is associated with both the development of ARDS and a known mechanism of VILI (Wang, Chai et al., 2019). Krenn et al. reviewed the impact of mechanical ventilation on RAS in the patients with ARDS with the intent of discovering novel biomarkers and possible therapeutic targets (Krenn et al.). Many clinical trials have been conducted using RAS-modifying drugs in mechanically ventilated ARDS patients and patients with other medical issues, that resulted in positive outcomes. These positive studies suggest RAS-modifying drugs maybe effective treatment of both the primary cause of ARDS as well as the secondary VILI that is associated with increased ARDS-related mortality.

ARDS can cause a massive release of inflammatory mediators often referred to as the “cytokine storm”. This hyperinflammatory response can lead to increase pulmonary microvascular permeability resulting in alveolar flooding with edema, a hallmark of ARDS lung pathology. There is growing evidence that cell-based therapies (mesenchymal stem cells) have therapeutic efficacy for ARDS (Lopes-Pacheco, Robba et al., 2020). Izrael et al. tested the treatment effect of human astrocytes therapy (AstroRx) in an endotoxin mouse ARDS model (Izrael et al.). They showed a significant reduction in multiple inflammatory mediators, improved lung histopathology and reduced mortality in AstroRx treated mice. This study demonstrated the immunosuppressive capacity of AstroRx cells and suggest an innovative ARDS treatment. In addition, this group is currently evaluating the therapeutic role of AstroRx in amyotrophic lateral sclerosis patients.

Impaired alveolar macrophage (AM) efferocytosis plays a role in ARDS pathogenesis, (Huang, Xiu et al., 2018), however, the ability to test AMs from ARDS patients is limited. Mahida et al. developed and *in vitro* model to assess the impact of ARDS on AMs (Mahida et al.). Normal AMs were harvested from lobectomy patients and then treated with bronchoalveolar lavage fluid (BALF) collected from ARDS patients. They found that ARDS BALF decreased AMs efferocytosis and Rac1 gene expression, but phagocytosis was not impaired. These findings are similar to that found in AMs from ARDS patients suggesting that this is an effective *in vitro* model to study the impact of ARDS on AMs. They also found that Rho-associated kinase partially resorted AM efferocytosis.

## Treatment strategies

Severe ARDS can lead to respiratory failure as well as hemodynamic collapse, caused by right ventricular (RV) failure. Clinical options at this point include extracorporeal membrane oxygenation (ECMO) giving the lung more time to heal. Petit et al. reviewed the pathophysiology of RV function in ARDS and some potential treatments to improve function including ventilator adjustments, prone positioning, nitric oxide (NO) inhalation, and veno-venous ECMO (VV-ECMO) (Petit et al.). The impact of ventilator settings on RV failure and acute cor pulmonale was reviewed extensively. It was concluded that ventilator settings should be adjusted to a Pplat <27cmH_2_O, inspiratory rate increased to reduce PaCO_2_, limit PEEP level, and optimize O_2_ delivery not the P/F ratio. They conclude that the RV protective approach should be evaluated in a future RCT with ECMO considered in extreme failure. Integrating the findings from this study to those by Andrews et al. and Rola and Daxon, pulmonary vascular resistance (PVR) is minimal at normal EELV (i.e., functional residual capacity) (Simmons et al., 1961). Thus, an important protective mechanism of the TCAV™ method may be to restore normal EELV, reduce PVR, which would improve RV function by decreasing afterload.

Few pharmacological approaches to treat ARDS are available. Liu and Dean reviewed the possible use of gene therapy that offers a highly controlled and targeted strategy to treat acute lung injury at the molecular level (Liu and Dean). Topics include delivery stategy, classes of targeted genes, and outcomes on ARDS pathogensis and resolution. They conclude that no combination of genes is responsible for ARDS, although several genes have been targeted up- and downregulation of genes with varying degrees of success. Early studies focused on increasing the expression of ion channels and tranporters to accelerate alveolar edema removal and have had minimal success. Current strategies targeting a reduced inflammatory response and repair or strengthening the alveolar-capillary barrier have shown more promise. Greater emphasis should be placed on studies using more clinically applicable large animal models designed to treat existing disease. The major limitation of effective gene therapy remains optimizing the gene delivery system.

Ultimately, treatment strategies for ALI and ARDS must be tested in RCTs to identify clinical efficacy. Villar et al. reviewed the physiologic-based gaps in 14 negative and positive RCTs (Villar et al.). Treatment strategies used in these RCTs included adjunct therapies (neuromuscular blockage (NMB) and prone position), optimal PEEP selection, HFOV, ECMO, and immune modulators. The findings from their study were as follows:

The ACURASYS trial investigated the hypothesis that by eliminating spontaneous breathing using NMB would improve lung mechanics and oxygenation in ARDS patients. However, it was concluded that NMB is not recommended in moderate to severe ARDS since patient-ventilator asynchronies increase. From a physiologic standpoint NMB should be used only if the patient has a ventilator pattern that could result in VILI.

Although the PROSEVA trial is controversial, the prone position has been shown to be highly effective as an ARDS treatment. There are 5-mechanisms suggested for the proning-induced improved oxygenation: (i) increased EELV, (ii) changes in regional diaphragm motion, (iii) improve ventilation/perfusion matching, (iv) increased secretion clearance, and (v) the weight of the heart is removed from the lung. A second clinical trial was suggested to confirm the large treatment effect seen in the first RCT.

Although there is strong physiologic evidence that heterogeneous lung collapse is a mechanism driving VILI there is currently no consensus on the use of RMs to open the lung nor the method to set PEEP to normalize EELV and stabilize alveoli. The ART and PHARLAP trials showed no benefit of RMs with the ART trial showing an increase in ARDS related 6-months mortality. The EPVent study using esophageal-guided PEEP showed no difference in mortality as compared with the control group. Both studies had numerous design faults and thus with fine tuning both may 1 day improve outcomes but currently neither strategy is recommended.

Similar results were found in the EOLIA trial using ECMO vs. a control group which was stopped early. The main problems with the study were the expected 20% absolute risk reduction was unreasonable and 28% of the patients in the control group crossed over to receive ECMO. Also like the above ART and EPVent studies another ECMO RCT was recommended with a lower anticipated absolute risk and no cross over patients.

Inflammation causing disruption to both pulmonary endothelium and epithelium resulting in a high permeability pulmonary edema is a hallmark of ARDS pathophysiology. Thus, RCTs to reduced inflammation and edema have been conducted. However, a systematic review showed that there is currently no pharmacologic intervention that could reduce ARDS mortality (Santacruz, Pereira et al., 2019). The BALTI-2 study tested salbutamol as a method to increase alveolar edema clearance and the HARP-2 tested the anti-inflammatory simvastatin. Salbutamol actually increased mortality and simvastatin had no effect but like many of the RCTs discussed above there were serious concerns on the quality of the data for multiple reasons. The INTEREST trial tested interferon (IFN) β, which was designed to upregulate CD73 preventing vascular leakage. There was no reduction in mortality but once again the study design was flawed. Lastly, the recent DEXA-ARDS trial for moderate to severe ARDS using dexamethasone decreased the risk of 60-days mortality by and absolute 15%, paving the way to using steroids in COVID-19 patients.

This group concludes that future RCTs must be personalized to subclasses of ARDS patients that are physiologically able to respond to the treatment therapy. In addition, treatment must be personalized to the patient’s specific lung physiology and morphology in order to improve patient outcomes.

## Lung fluid balance

Inflammation and biotrauma can increase the permeability of air-blood barrier (ABB) resulting in pulmonary edema and plays a major role in both ARDS and VILI pathogenesis. (Del Sorbo, Goligher et al., 2017; Matthay, Zemans et al., 2019). Beretta et al. computationally modeled ABB disruption taking into account Starling forces, surfactant function, and edema safety factors including: (i) capacity of fluid accumulation on the thick side of the ABB, (ii) increased interstitial pressure, and (iii) increased lymph flow (Beretta et al.). This extensive review covers the mechanisms of ABB disruption, edema development time constants, factors preventing the development of edema, a “tipping point” in ABB injury followed by rapid alveolar flooding, and the role of spontaneous and mechanical ventilation. This study supports those of Rola and Daxon study (Rola and Daxon) showing that the TCAV™ method to set and adjust the APRV mode would favor reduced edema formation.

## Conclusion

The papers in our Research Topic discuss novel and innovative treatment strategies for the patient with acute lung injury (ALI). Potential breakthrough methods of protective mechanical ventilation are discussed. Although there are currently no pharmacologic therapies for ARDS the recent DEXA-ARDS trial lends hope that pharmacologic interventions may be available in the near future. The mechanisms behind ALI-induced loss of lung fluid balance resulting in edema formation are discussed and analyzed using computational models. This work ties in with novel ventilation strategies that may also reduce edema formation. In addition, the innovative use of gene therapy to remove edema from flooded alveoli is reviewed. Lastly, success or failure of RCTs designed to treat ARDS are analyzed form physiologic basis with the goal to identify which clinical trials should be repeated and how to improve the quality of future RCTs.
